# Allosteric modulation of integral protein activity by differential stress in asymmetric membranes

**DOI:** 10.1093/pnasnexus/pgad126

**Published:** 2023-04-11

**Authors:** Paulina Piller, Enrico F Semeraro, Gerald N Rechberger, Sandro Keller, Georg Pabst

**Affiliations:** Biophysics, Institute of Molecular Bioscience (IMB), NAWI Graz, University of Graz, Graz 8010, Austria; BioTechMed Graz, Graz 8010, Austria; Field of Excellence BioHealth—University of Graz, Graz 8010, Austria; Biophysics, Institute of Molecular Bioscience (IMB), NAWI Graz, University of Graz, Graz 8010, Austria; BioTechMed Graz, Graz 8010, Austria; Field of Excellence BioHealth—University of Graz, Graz 8010, Austria; Field of Excellence BioHealth—University of Graz, Graz 8010, Austria; Biochemistry, Institute of Molecular Bioscience (IMB), NAWI Graz, University of Graz, Graz 8010, Austria; Omics Center Graz, BioTechMed Graz, Graz 8010, Austria; Biophysics, Institute of Molecular Bioscience (IMB), NAWI Graz, University of Graz, Graz 8010, Austria; BioTechMed Graz, Graz 8010, Austria; Field of Excellence BioHealth—University of Graz, Graz 8010, Austria; Biophysics, Institute of Molecular Bioscience (IMB), NAWI Graz, University of Graz, Graz 8010, Austria; BioTechMed Graz, Graz 8010, Austria; Field of Excellence BioHealth—University of Graz, Graz 8010, Austria

**Keywords:** lateral pressure profile, lipid–protein interactions, phospholipase

## Abstract

The activity of integral membrane proteins is tightly coupled to the properties of the surrounding lipid matrix. In particular, transbilayer asymmetry, a hallmark of all plasma membranes, might be exploited to control membrane-protein activity. Here, we hypothesized that the membrane-embedded enzyme outer membrane phospholipase A (OmpLA) is susceptible to the lateral pressure differences that build up between such asymmetric membrane leaflets. Upon reconstituting OmpLA into synthetic, chemically well-defined phospholipid bilayers exhibiting different lateral pressure profiles, we indeed observed a substantial decrease in the enzyme’s hydrolytic activity with increasing membrane asymmetry. No such effects were observed in symmetric mixtures of the same lipids. To quantitatively rationalize how the differential stress in asymmetric lipid bilayers inhibits OmpLA, we developed a simple allosteric model within the lateral pressure framework. Thus, we find that membrane asymmetry can serve as the dominant factor in controlling membrane-protein activity, even in the absence of specific, chemical cues or other physical membrane determinants such as hydrophobic mismatch.

**Significance Statement**

All plasma membranes of biological cells display an asymmetric distribution of lipids that is widely believed to be essential for cellular function. However, a quantitative understanding of the physiological benefits of membrane asymmetry is sparse. In particular, it is unknown how membrane asymmetry controls the activity of integral membrane proteins. Reconstituting outer membrane phospholipase A, a well-characterized integral membrane enzyme, into lipid membranes, we find direct experimental evidence for an allosteric modulation of protein activity by differential stresses stored in asymmetric bilayers. The generic nature of our findings suggests that analogous mechanisms are likely to be at play with many other plasma membrane proteins.

Lipids of cellular plasma membranes distribute asymmetrically across leaflets (1, 2). This lipid asymmetry can lead to distinct structural and dynamic membrane properties, such as, e.g. thickness, lipid packing, elasticity, or fluidity (3–6) with important consequences for the activity of peptides and proteins that interact with or reside within lipid membranes. For instance, the bacterial toxin perfringolysin O forms pores in symmetric but not in asymmetric lipid bilayers (7). Along the same lines, the nicotinic acetylcholine receptor partitions into lipid nanodomains only if sphingomyelin is enriched in the outer leaflet (8). A further prominent example are mechanosensitive channels, which respond to changes in the lateral pressure profile across a lipid bilayer that are due to asymmetric lipid distributions (9). Likewise, the oligomerization of membrane proteins has been inferred to be affected by the lateral pressure profile and, thus, by membrane asymmetry (10), and the Na${}^{+}$/K${}^{+}$-ATPase has been suggested to respond to the asymmetric distribution of charges in mammalian plasma membranes (11, 12). In general, membrane-protein folding and topology are intimately linked to lipid distribution (13). Still, only little is understood about the effects of membrane asymmetry on the functions of integral membrane enzymes.

Thus, we aimed to quantitatively decipher the influence of membrane asymmetry on the activity of an integral membrane enzyme that is expected to be responsive to changes in membrane asymmetry. For this purpose, outer membrane phospholipase A (OmpLA) appeared to be a particularly well-suited candidate. OmpLA resides in the highly asymmetric outer membrane of the bacterium *Escherichia coli*, where it folds into a 12-stranded antiparallel β-barrel. Upon dimerization, OmpLA hydrolyzes phospholipids, which are thus removed from the outer leaflet of the outer membrane (14). In vivo, OmpLA has been suggested to be in a dormant monomeric state in its native asymmetric environment but activated upon severe perturbation of the outer membrane, for example, by membrane-active toxins (14). Once phospholipids have entered the outer membrane leaflet, OmpLA homodimers might then be stabilized predominantly by interactions with the acyl chain of the lipid substrate (15). In vitro, Ca${}^{2+}$ can be used to control protein activity even in symmetric lipid bilayers (16), presumably because Ca${}^{2+}$ is required to mutually align the active sites located in two dimerizing OmpLA monomers.

However, it has remained unclear whether OmpLA is activated mainly through such specific, chemical interactions or rather through physical changes in the bulk properties of the membrane, such as alterations in membrane asymmetry. We thus hypothesized that the loss of lipid asymmetry associated with membrane perturbation might contribute to the activation of OmpLA. In particular, we wondered whether changes in OmpLA activity upon altering membrane asymmetry could be rationalized in terms of a simple allosteric model that couples enzyme activity to changes in lateral pressure profile (Fig. 1).

**Fig. 1.**
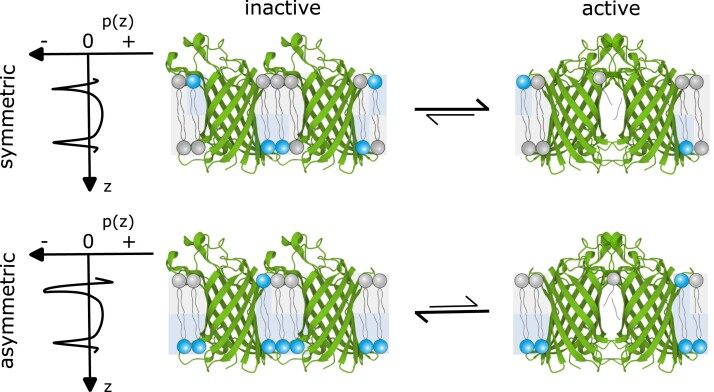
Schematic of allosteric coupling of OmpLA activity to the lateral pressure profiles, p(z), in symmetric and asymmetric bilayers composed of a binary lipid mixture (Protein Data Bank codes: 1QD5, 1QD6 (16)). Chemically different lipid species are indicated by the color of the headgroup.

For testing our hypothesis, we interrogated the activity of OmpLA in asymmetric vesicles composed of 1-palmitoyl-2-oleoyl-sn-glycero-3-phosphocholine (POPC) and 1-palmitoyl-2-oleoyl-sn-glycero-3-phosphoethanolamine (POPE). Neither of these two lipids has been reported to interact specifically with OmpLA. Moreover, while POPC is cylindrically shaped, POPE is best described by a cone-like molecular shape (17, 18), leading to distinct lateral pressure profiles in bilayers. Consequently, we reasoned that we could dissect the influence of generic membrane properties on the enzymatic activity of OmpLA by varying the POPC/POPE leaflet composition of synthetic lipid bilayers. Indeed, we observed a decrease in the phospholipid hydrolysis rates upon increasing membrane asymmetry. Strikingly, the slowing down of lipid degradation amounted to up to two orders of magnitude, almost completely inhibiting the enzyme. Instead, OmpLA was equally active in all symmetric mixtures of the same lipids. Simple theoretical considerations on protein shape and differential stress in asymmetric bilayers agree with these experimental findings over a large range of studied membrane compositions revealing potential generic avenues for integral proteins to harness membrane asymmetry.

## Results

**Fig. 2.**
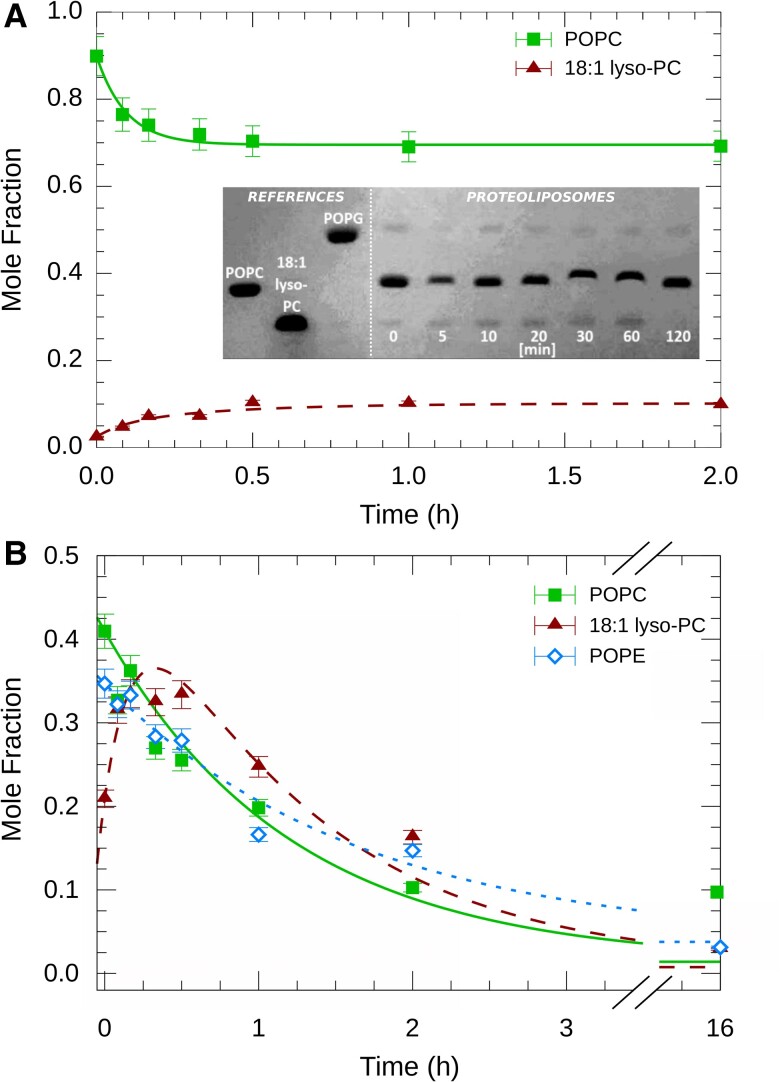
A) Time-resolved HPTLC showing OmpLA activity in proteoliposomes having either symmetric bilayers made from POPC only (panel A) or asymmetric bilayers with POPC in the inner leaflet and mostly (∼88%) POPE in the outer leaflet (panel B). The lipid composition before OmpLA activation is detailed in Tables S1 and S2. The insert to panel A depicts a representative HPTLC plate showing a time series taken at different points after the addition of ${\mathrm{Ca}}^{2+}$. Lines in panel B show fits of the hydrolysis kinetics (see the main text).

We started by generating POPC proteoliposomes using established protocols (19). All vesicles were doped with 1-palmitoyl-2-oleoyl-sn-glycero-3-phospho-($1^{\prime }$-rac-glycerol) (POPG) to ensure the formation of unilamellar vesicles (20). Functional refolding of OmpLA into the ∼100-nm large unilamellar vesicles was tested using a colorimetric assay based on the hydrolysis of 2-hexadecanoylthio-1-ethyl-phosphorylcholine (HEPC) (21). We observed a linear increase in specific activity with the protein/lipid molar ratio, $x_{O}=[OmpLA]/[Lipid]$, (Fig. S3). Further, time-resolved high performance thin layer chromatography (HPTLC) (in the absence of HEPC) showed a monotonous increase in the concentration of 18:1 lyso-PC over time and a concomitant decrease in the concentration of POPC, confirming the enzyme’s hydrolytic activity (22) (Fig. 2A); see also Fig. S4.

Next, we exchanged a large fraction of the POPC molecules in the outer leaflet of proteoliposomes with POPE using cyclodextrin-mediated lipid exchange, which is a well-established technique for producing asymmetric lipid vesicles (see, e.g. (23, 24, 3, 5–7)); details are given in the Supplementary material. HPTLC measurements of the asymmetric proteoliposomes prior to the activation of OmpLA indicated an exchange efficiency of ∼88% of the outer leaflet (Table S1). Interestingly, we now observed much slower lipid hydrolysis rates in these asymmetric POPE/POPC proteoliposomes as compared with symmetric POPC proteoliposomes (Fig. 2). Note that the slower kinetics and, thus, the longer timescale of this experiment sufficed to show also the hydrolysis of 18:1 lyso-PC. Complementary ultra performance liquid chromatography-mass spectrometry (UPLC-MS) experiments confirmed these observations and revealed the presence of additional hydrolysis products (Fig. S5).

For quantitative insight, kinetic HPTLC data were analyzed in terms of rate equations (Eqs. 8–9). In the following, we focus on the normalized hydrolysis rates ${\bar{k}}_{i}=k_{i}/x_{O}$ (i=POPC;POPE;18:1lyso−PC) to account for differences in OmpLA concentration within proteoliposomes (Tables S3, S4), which is validated by the linear increase of specific activity with $x_{O}$ (Fig. S3B); but see also Horrevoets et al. (25). Nota bene, in the case of POPC and POPE ${\bar{k}}_{i}$ refers to the combined hydrolysis of both hydrocarbons.

Fitting HPTLC data of symmetric POPC proteoliposomes (Fig. 2A) with Eq. (9), we found ${\bar{k}}_{\mathrm{POPC}}∼0.73\;s^{-1}$. Instead, rates dropped to ${\bar{k}}_{\mathrm{POPE}}\approx {\bar{k}}_{\mathrm{POPC}}∼0.1\;s^{-1}$ for the activity of OmpLA in asymmetric POPC/POPE bilayers. To further substantiate our findings on the different activity of OmpLA in symmetric and asymmetric membranes, we produced proteoliposomes with various degrees of asymmetry, that is, different percentages of POPE molecules in the outer leaflet. This was achieved by first reconstituting OmpLA in POPC/POPE mixtures with different molar ratios, ranging from 5.3 to 1.7 (Table S1). This was followed by outer leaflet exchange with POPE as detailed in the Materials and Methods section. Assuming that only outer leaflet composition is modified yields asymmetries of POPC molar fraction in outer and inner leaflets, $x_{\mathrm{POPC}}^{\mathrm{out}}-x_{\mathrm{POPC}}^{\mathrm{in}}$, from −0.92 to −0.37 (Table 1). Additionally, we reconstituted OmpLA into three differently composed symmetric vesicles; POPE and two POPC/POPE mixtures ($x_{\mathrm{POPC}}∼0.44$, $x_{\mathrm{POPC}}∼0.52$, see Table 1) corresponding to the highest and lowest POPE levels achieved in our asymmetric proteoliposomes (Table S2). Based on our hypothesis (Fig. 1), these symmetric proteoliposomes served as negative controls.

**Table 1.** OmpLA activity in symmetric and asymmetric membranes.

Composition${}^{a}$		${\bar{k}}_{i}$ (s${}^{-1}$)${}^{b}$			$J_{0b}$ (nm${}^{-1}$)${}^{c}$
$x_{\mathrm{POPC}}^{\mathrm{in}}$	$x_{\mathrm{POPC}}^{\mathrm{out}}$	POPC	POPE	18:1 lyso-PC	
1.00		0.73	–	0.16	0
0.53		0.53	0.61	0.90	0
0.51		0.49	0.66	0.88	0
0.45		0.87	0.20	1.01	0
0.43		0.65	0.33	0.48	0
0.00		–	1.29	–	0
1.00	0.08	0.08	0.06	0.45	− 0.147
1.00	0.13	0.08	0.10	0.68	− 0.140
0.75	0.15	0.004	0.014	0.13	− 0.098
0.85	0.24	0.04	0.06	0.44	− 0.097
0.85	0.25	0.05	0.06	0.80	− 0.096
0.75	0.21	0.004	0.016	0.11	− 0.088
0.65	0.24	0.06	0.07	0.45	− 0.067
0.65	0.28	0.21	0.23	3.9	− 0.061

${}^{a}$ Molar fraction of POPC within a given leaflet. The first six rows correspond to symmetric POPC/POPE mixtures; numbers correspond to the overall molar fraction of POPC.

${}^{b}$ Uncertainties for ${\bar{k}}_{i}$ vary between 10 and 30%.

${}^{c}$ Calculated rigidity-weighted spontaneous bilayer curvature (uncertainties vary between 10 and 30%).

Table 1 summarizes the hydrolysis rates for all studied symmetric and asymmetric proteoliposomes, including repeats on independently prepared samples. No systematic changes in hydrolysis rates with lipid composition were observed in our negative control samples of symmetric membranes (see also Figs. S7–S9). Variations of ${\bar{k}}_{i}$ rather reflect experimental uncertainties. The average hydrolysis rate was ∼0.7 s${}^{-1}$. In stark contrast, lipid hydrolysis proceeded much slower in all asymmetric proteoliposomes (Figs. S8). Accordingly, phospholipid hydrolysis rates were up to two orders of magnitude lower than in symmetric bilayers. Moreover, ${\bar{k}}_{i}$ decreased with increasing membrane asymmetry.

## Discussion

To rationalize our findings, it is instructive to first compare the activity of OmpLA to that of other lipases. For example, the bovine-brain phospholipase A${}_{1}$ hydrolyzes, depending on the substrate, between 30 and 300 lipids/s (26). In stark contrast, our activity measurements in symmetric vesicles yield ∼0.56 lipids/s for each active OmpLA dimer. We speculate that this much slower hydrolysis rate results from the requirement for OmpLA to form dimers with appropriately aligned active sites.

Following this line of reasoning, it is then likely that the probability of aligning the active sites of two freely diffusing OmpLA monomers depends on the bulk properties of the surrounding membrane, such as hydrophobic thickness and internal stress. Indeed, the hydrophobic mismatch between transmembrane domains and bilayer thickness has been demonstrated to promote aggregation of integral proteins in order to reduce the overall free-energy cost incurred by membrane deformation (27). However, we observed a similar activity of OmpLA in all symmetric proteoliposomes (Table 1), although POPE bilayers are thicker than POPC membranes (28, 29). Moreover, the phospholipid hydrolysis rate decreased with increasing POPE asymmetry and, hence, increased hydrophobic mismatch, ruling out hydrophobic mismatch as a significant determinant of OmpLA activity. Notably, the distinct activities of OmpLA in asymmetric and symmetric control vesicles also clearly rule out a rapid loss of asymmetry, as previously reported for membrane-active α-helical amphipathic peptides (30). The same study also showed that gramicidin A, which is more stably anchored in bilayers than amphipathic α-helical peptides, accelerates lipid flip-flop to a much lesser extent. We speculate that the rigid β-barrel conformation of OmpLA similarly results in negligible lipid scrambling, at least before its activation with Ca${}^{2+}$.

### Allosteric model

Excluding hydrophobic matching, we thus considered a different scenario in which the enzymatic activity of OmpLA is modulated by changes in the lateral pressure profile upon altering the degree of membrane asymmetry (31). Within this conceptual framework, conformational changes of a membrane-embedded protein that modify its overall size or geometry need to take place against an external field of lateral pressure. Importantly, the lateral pressure profile depends on lipid composition and can be quantified experimentally in terms of the intrinsic lipid curvature and bending modulus (32). The bending-rigidity-weighted spontaneous curvature of the asymmetric bilayers $J_{0b}$ (4) is a convenient way to quantify this stress. Negative $J_{0b}$ indicates a preference for concave membrane curvatures, that is, outward bending, and vice versa for positive $J_{0b}$. Table 1 reports $J_{0b}$ values for all studied asymmetric membranes using published data for POPE and POPC (17, 18, 33) (see the Materials and Methods section). All asymmetric membranes indicated a predisposition for concave membrane curvatures, reaching $J_{0b}=-0.147$ nm${}^{-1}$ at the highest POPC/POPE transbilayer asymmetry.

To derive the effect of this differential stress on OmpLA activity, we assumed that the protein population is divided into predominantly active (*A*) and inactive (*N*) states. Note that both states, *A* and *N*, refer to Ca${}^{2+}$-stabilized dimers, which, however, differ in terms of their hydrolysis rates. Assuming the inactive state to be enzymatically silent enables us to write the rate of hydrolysis of a given phospholipid by OmpLA-dimers as $k_{i}={\tilde{k}}_{i}x_{A}$, where $x_{A}=[A]/([A]+[N])$ is the molar fraction of states *A* and ${\tilde{k}}_{i}$ is the lipid specific hydrolysis rate per unit fraction of *A*.

By definition, the equilibrium constant between states A⇌N is $K=[A]/[N]=x_{A}/(1-x_{A})$ and the free energy of activation of the enzyme $\Delta G^{*}=-k_{B}T\ln\;K$; with $k_{B}$ being Boltzmann’s constant and *T* the absolute temperature. In order to link $\Delta G^{*}$ to the work that the pressure field exerts on the enzymes, we write for the chemical potential of given state s=A,N of OmpLA


(1) $$\mu_{s}=\mu_{s}^{*}+k_{B}T\ln\;x_{s}-W_{s},$$

where $\mu_{s}^{*}$ is the standard chemical potential and $W_{s}$ is the work associated to the internal lateral pressures (31). At thermodynamic equilibrium $\mu_{A}=\mu_{N}$, independent of whether the membrane is symmetric or asymmetric. Assuming that $\mu_{A}^{*}-\mu_{N}^{*}$ is equal for symmetric and asymmetric bilayers, it follows that


(2) $$\begin{array}{rl} & \underset{\Delta G^{*,sym}}{\underbrace{-k_{B}T\ln\;K^{sym}}}+\underset{\Delta W^{sym}}{\underbrace{{\left(W_{A}-W_{N}\right)}^{sym}}}\\ & \;=\underset{\Delta G^{*,asym}}{\underbrace{-k_{B}T\ln\;K^{asym}}}+\underset{\Delta W^{asym}}{\underbrace{{\left(W_{A}-W_{N}\right)}^{asym}}}, \end{array}$$

which simplifies to


(3) $$\Delta G^{*,sym}-\Delta G^{*,asym}=-\Delta W^{sym}+\Delta W^{asym}=-\Delta W.$$

That is, the difference in the activation free energy of OmpLA between symmetric and asymmetric bilayers is given by the associated difference of work, ΔW, that needs to be performed against lateral pressures.

Next, in the spirit of an allosteric model of protein activity, we assume that the total hydrolysis rate depends only on the number of active enzymes but not on membrane asymmetry. Hence, ${\tilde{k}}_{i}^{\mathrm{sym}}={\tilde{k}}_{i}^{\mathrm{asym}}$. It follows that the ratio of lipid hydrolysis rates in asymmetric and symmetric membranes just depends on the ratio of active proteins in either system $k_{i}^{\mathrm{asym}}/k_{i}^{\mathrm{sym}}≃x_{A}^{\mathrm{asym}}/x_{A}^{\mathrm{sym}}$. Using Eq. (3), we thus obtain


(4) $$k^{asym}=k^{sym}\frac{e^{\Delta G^{*,sym}/k_{B}T}+1}{e^{(\Delta G^{*,sym}+\Delta W)/k_{B}T}+1}.$$

Inspection of Eq. (4) shows that $k_{i}^{\mathrm{asym}}<k_{i}^{\mathrm{sym}}$ for $\Delta W/k_{B}T>0$. That is, the phospholipid hydrolysis rate is predicted to decrease in asymmetric membranes because of increasing contributions from differential stress.

### Estimation of differential stress

Evaluation of Eq. (4) requires knowledge about ΔW and $\Delta G^{*,\mathrm{sym}}$. Focusing on the first parameter, we write the following equation (31):


(5) $$\Delta W=-\int dzA(z)\Delta p(z)\approx -\sum_{i=1,2}\sum_{\;j=\mathrm{out},\mathrm{in}}a_{i}^{j}\Delta p_{i}^{j},$$

where *z* is the transbilayer coordinate, A(z) is the depth-dependent cross-sectional protein area profile, and Δp(z) is the lateral pressure differences between symmetric and asymmetric bilayers. The approximation of the integral at the right-hand side of the above equation assumes a rotationally symmetric protein shape, allowing us to estimate ΔW based on experimentally accessible parameters (vide infra). Here, $a_{i}^{\mathrm{out}/\mathrm{in}}$ are the coefficients of a Taylor expansion of A(z) in outer and inner leaflets and $\Delta p_{i}^{\mathrm{out}/\mathrm{in}}$ refers to the difference of the moments of the lateral pressure profiles between symmetric and asymmetric bilayers for each leaflet (10, 34). The active form of OmpLA is a homodimer (15), whose shape can be roughly approximated by an hourglass form using crystallographic data (16) yielding $a_{1}^{\mathrm{out}/\mathrm{in}}=0.344\pm 0.014$ nm and $a_{2}^{\mathrm{out}/\mathrm{in}}=(2.58\pm 0.16)\times 10^{-3}$ (see the Materials and Methods section).

The first and second moments of the lateral pressure profile depend on several leaflet properties, such as the spontaneous curvature $J_{0}^{\mathrm{out}/\mathrm{in}}$, bending rigidity $\kappa_{m}^{\mathrm{out}/\mathrm{in}}$, Gaussian curvature modulus $\kappa_{G}^{\mathrm{out}/\mathrm{in}}$, and the position of the neutral plane $h^{\mathrm{out}/\mathrm{in}}$ (32). In compositionally asymmetric membranes, these parameters will differ for each leaflet leading to differential stress between the two membrane halves (Table 1). Recently, Hossein and Deserno (4) pointed at a second source of differential stress in asymmetric membranes originating from a lateral area mismatch between the membrane leaflets. This can be to first order accounted for in the first moment of the lateral pressure profile, yielding


(6) $$p_{1}^{\mathrm{out}/\mathrm{in}}≃\pm \;\alpha \kappa_{m}^{\mathrm{out}/\mathrm{in}}\;J_{0}^{\mathrm{out}/\mathrm{in}},$$

where the correction factor α=15.5/14 accounts for the differential stress induced by area mismatch in flat asymmetric bilayers (4); the sign in Eq. (6) is negative for the inner leaflet. Note that α=1 in absence of differential stress, i.e. in symmetric systems. The second moment of the lateral pressure profile for each leaflet is (32)


(7) $$p_{2}^{\mathrm{out}/\mathrm{in}}=2h^{\mathrm{out}/\mathrm{in}}\alpha \kappa_{m}^{\mathrm{out}/\mathrm{in}}\;J_{0}^{\mathrm{out}/\mathrm{in}}-\kappa_{G}^{\mathrm{out}/\mathrm{in}}.$$

Both moments of the lateral pressure profile were estimated using previously reported structural and elastic data, as detailed in the Materials and Methods section. The resulting estimates for ΔW upon application of Eq. (5) report a significant dependance on membrane asymmetry, with values up to ∼10 $k_{B}T$.

### Matching the model with OmpLA activity

**Fig. 3.**
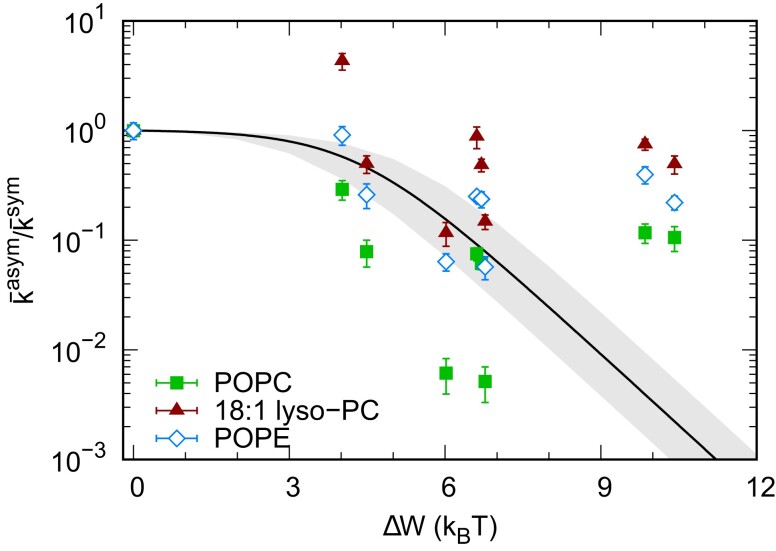
Ratio of normalized hydrolysis rates, ${\bar{k}}_{i}$, between asymmetric and symmetric proteoliposomes (see Table 1): POPC (squares), 18:1 lyso-PC (triangles), and POPE (diamonds). The curve shows a global fit based on Eq. 4, and the gray band depicts the 99% confidence interval. Reference rates at ΔW≃0 are the averages from symmetric proteoliposomes at different POPC/POPE ratios (see Table 1). Uncertainties for ΔW are in the range 20–40%.

In order to test whether our allosteric model is able to account for the decrease of OmpLA activity with increasing asymmetry, we jointly fitted the hydrolysis rates of all lipids by assuming a single $\Delta G^{*,\mathrm{sym}}$ applying Eq. (4). Our model was able to reproduce the overall decrease of phospholipid hydrolysis rates with increasing membrane asymmetry ΔW (Fig. 3) with $\Delta G^{*,\mathrm{sym}}=-4.3\pm 0.3$$k_{B}T$, indicating that the conformational equilibrium is on the active side in all symmetric membranes. The large discrepancy between our model and experimental data at ΔW>7$k_{B}T$ may be due to various contributions that are difficult to control experimentally or account for theoretically. For example, lipid scrambling, which most likely goes in hand with the hydrolytic activity of the protein, will continuously decrease ΔW. This variation is expected to be stronger the higher the initial membrane asymmetry.

All experiments were also performed in the absence of Ca${}^{2+}$ (Figs. S7B, D, F and S8B, D, F, H). OmpLA displayed some basal activity, but the overall hydrolysis rate slowed down. Interestingly, however, the normalized rates also decreased with increasing differential stress (Fig. S9).^1^ That is, Ca${}^{2+}$ merely increases the overall hydrolysis rates but does not affect the allosteric mechanism as such. This agrees with Ca${}^{2+}$ contributing little to the stabilization of OmpLA dimers (15) and supports the idea that Ca${}^{2+}$ is unlikely to be involved in the regulation of the enzyme in its natural environment (14). Instead, the differential stress resulting from membrane asymmetry emerges as a decisive determinant controlling the enzymatic activity of OmpLA.

### Conclusion

We provide experimental evidence for the functional coupling of the activity of the integral membrane enzyme OmpLA to membrane asymmetry. Our simple theoretical framework based on protein allostery is able to reproduce essential features of OmpLA activity under differential membrane stress. Specifically, changes in phospholipid hydrolysis rates are correctly predicted to be negligible for $\Delta W<\Delta G^{*,\mathrm{sym}}$ but asymptotically decrease as $e^{-\Delta W/k_{B}T}$ for $\Delta W>\Delta G^{*,\mathrm{sym}}$. Future refinements of our allosteric model might take into account interactions of OmpLA with charged phospholipids, and lipopolysaccharides, which have been shown to modulate the structure and dynamics of the extracellular loop of OmpLA (35). On more general grounds, our study demonstrates how membrane-embedded proteins can exploit the differential stress stored in asymmetric cellular membranes, encouraging further research in this direction.

## Materials and methods

### Lipids and chemicals

POPC, POPE, and POPG were purchased from Avanti Polar Lipids (Alabaster, AL, USA). HEPC was acquired from Cayman Chemical (Ann Arbor, MI, USA); see the Supplementary material for additionally used chemicals and solvents.

### Protein production, purification, and reconstitution

OmpLA expressed in *E. coli*, purified, refolded, and reconstituted in symmetric lipid vesicles of defined composition following previously established protocols (19). For details see the Supplementary material.

### Preparation of asymmetric proteoliposomes

Asymmetric proteoliposomes were prepared using methyl-β-cyclodextrin following the heavy donor exchange protocol (24). Here, proteoliposomes were defined as acceptor vesicles, analogous to Markones et al. (36). Donor multilamellar vesicles were composed of POPE; see Figs. S1 and S2 in the Supplementary material for further details.

### Colorimetric enzyme activity assay

The enzymatic activity of OmpLA was assessed by following a standard phospholipase assay based on the OmpLA-catalyzed hydrolysis of HEPC (21); see the Supplementary material for further details.

### High performance thin layer chromatography

Lipids were extracted from the proteoliposomes using the Folch method (37). Samples were spotted onto silica plates (Sigma-Aldrich, Steinheim, Germany) with the automatic TLC sampler 4 (CAMAG, Muttenz, Switzerland) and developed and analyzed as detailed in the Supplementary material.

### Ultra performance liquid chromatography mass spectrometry

Additionally, selected lipid extracts from proteoliposomes were analyzed by UPLC-MS. Lipid components were chromatographically separated using a 1290-UHPLC system (Agilent, Waldbronn, Germany) and subsequently injected into a 4670 triple quadrupole mass spectrometer (Agilent, Waldbronn, Germany) equipped with an electrospray ionization source. Details for the applied procedures are given in the Supplementary material.

### Analysis of OmpLA activity

Time-resolved HPTLC data for the hydrolysis of POPC, POPE, and 18:1 lyso-PC can be described empirically by the rate equations


(8) $$\begin{array}{cc} & \;\;\dot{\left[P\right]}(t)=-(k_{1}+k_{2})[P](t)=-k_{P}[P](t)\\ & \dot{\left[L_{2}\right]}(t)=k_{1}[P](t)-k_{L}[L_{2}](t), \end{array}$$

where [P] is the concentration of POPC or POPE, $\left[L_{2}\right]$ is the concentration of 18:1 lyso-PC, $k_{1}$ is the hydrolysis rate of *sn-1* hydrocarbons, $k_{2}$ is the hydrolysis rate of *sn-2* hydrocarbons, and $k_{L}$ is the hydrolysis rate of $\left[L_{2}\right]$. Given the boundary conditions for t=0 and t→∞, the solutions of Eq. 8 are


(9) $$\begin{array}{cc} \left[P\right](t) & \;\;\;\;=\;\left([P](0)-[P{]}_{\infty }\right)e^{-k_{P}t}+[P{]}_{\infty }\\ \left[L_{2}](t\right) & \;\;\;\;=\;\left([L_{2}](0)-[L_{2}{]}_{\infty }\right)e^{-k_{L}t}\\ & \;\;\;\;+k_{1}\frac{[P](0)-[P{]}_{\infty }}{k_{L}-k_{P}}\left(e^{-k_{P}t}-e^{-k_{L}t}\right)+[L_{2}{]}_{\infty }. \end{array}$$

Table 1 reports the $k_{P}$ and $k_{L}$ values from this analysis. Our analysis revealed a poor confidence for $k_{1}$, especially in the case of very fast hydrolysis. We thus used the $k_{P}$-values obtained from the [P](t) fits and constrained $k_{1}$ values in the range $\left[0\;:\;k_{P}\right]$. Table S5 lists the obtained results.

### Estimation of OmpLA geometrical parameters, average membrane rigidity, and lipid curvatures

The atomistic structure of active OmpLA homodimers (16) was approximated by a *z*-symmetric hourglass shape with an average central radius $R_{0}=1.91\pm 0.02$ nm and tilt angle $\phi^{\mathrm{out}}=\phi^{\mathrm{in}}=0.0286\pm 0.0009$ (Fig. S6). This yields for the Taylor coefficients (Eq. 5) $a_{1}^{\mathrm{out}/\mathrm{in}}=2\pi R_{0}\tan\;\phi^{\mathrm{out}/\mathrm{in}}=0.344\pm 0.014$ nm and $a_{2}^{\mathrm{out}/\mathrm{in}}=\pi \tan^{2}\;\phi^{\mathrm{out}/\mathrm{in}}=(2.58\;\pm 0.16)\times 10^{-3}$. Errors are estimates originating from uncertainties in ascribing the protein structure with a simple geometric shape.

First and second moments of the lateral pressures (Eqs. 6, 7) were calculated using previously reported estimates for spontaneous monolayer curvatures of POPE and POPC (17, 18). Nonlinear mixing due to the different PE/PC headgroup was considered in calculating molecular averages of spontaneous curvatures using the lipid-head PC/PE arc-ratio of 1.12 (18). Monolayer bending rigidity values were taken from Venable et al. (33) and averaged according to leaflet composition. These values were additionally multiplied by a factor of 1.5, based on most recent bending rigidity data for POPE/POPC asymmetric membranes (38).

The position of the neutral plane was estimated from structural data of POPC and POPE bilayers (28, 29), assuming that *h* coincides with the position of the lipid backbone (39). Gaussian curvature moduli were estimated using $\kappa_{G}\approx -0.8\kappa_{m}$ (40).

## Supplementary material

Supplementary material is available at *PNAS Nexus* online.

## Supplementary Material

pgad126_Supplementary_Data

## Data Availability

All data is included in the manuscript and Supplementary material.

## Note

1 Because of unknown contributions from OmpLA monomers, the obtained $\Delta G^{*,\mathrm{sym}}∼-6$$k_{B}T$ should not be compared to the activation free energy in the presence of calcium.
