# Supplementary material for: Allosteric modulation of integral protein activity by differential stress in asymmetric membranes
Source: PNAS Nexus. 2023 Apr 11;2(5):pgad126. doi: 10.1093/pnasnexus/pgad126 (PMC10153742; doi:10.1093/pnasnexus/pgad126)
Supplement: pgad126_Supplementary_Data [file pgad126_supplementary_data.pdf]

Supporting Information for

Allosteric Modulation of Integral Protein Activity by Differential Stress in Asymmetric Membranes

Paulina Piller, Enrico F. Semeraro, Gerald N. Rechberger, Sandro Keller, Georg Pabst

Corresponding Georg Pabst.
E-mail: georg.pabst@uni-graz.at

This PDF file includes:

Supporting text
Figs. S1 to S9
Tables S1 to S5
SI References

Supporting Information Text

Solvents and Chemicals. $CHCl_3$, MeOH, EtOH, tris(hydroxy-methyl) aminomethane (Tris), NaCl, $CaCl_2$, $MgCl_2$, $MnCl_2$, D(+)-saccharose, isopropyl-β-d-thiogalacto-pyranoside (IPTG), 5,5‘-dithiobis-(2-nitrobenzoic acid) (DTNB), urea, glycine, peptone, yeast extract, H_2SO_4 (96 % v/v), $Na_2S_2O_5$ and Na_2SO_3 were obtained from Carl Roth (Karlsruhe, Germany). Methyl-β-cyclodextrin (mβCD) and lauryldimethylamin-N-oxid (LDAO), $HClO_4$ (67−72 % v/v) and ammonium heptamolybdate tetrahydrate were acquired from Sigma-Aldrich (Vienna, Austria), ethylendiaminetetraacetic acid (EDTA), 1-amino-2-hydroxy-naphthalin-4-sulfonic acid (ANSA) and KH_2PO_4 from Merck (Darmstadt, Germany).

For ultra performance liquid chromatography mass spectrometry (UPLC-MS) measurements all solvents were at least HPLC grade. Water, 2-propanol, and phosphoric acid were purchased from Carl Roth (Karlsruhe, Germany), methanol from J.T.Baker (Austin, TX, USA), formic acid from Sigma-Aldrich (Vienna, Austria) and ammonium acetate was purchased from Merck (Darmstadt, Germany).

Protein Production and Purification. OmpLA without signal sequence was expressed in *Escherichia coli* BL21(DE3) cells (Novagen, Merck, Darmstadt, Germany) as inclusion bodies (IBs) utilizing a pET-24a (+) expression vector (Merck, Darmstadt, Germany). After inoculation of the lysogeny broth medium with *E. coli* BL21(DE3) cells, the production of OmpLA was induced by the addition of IPTG (0.4 mM final concentration). Incubation lasted for 3 h at 37 °C under agitation. Cells were harvested, washed and resuspended in 1:10 (wt/vol) ice-cold breakage buffer (50 mM Tris, 40 mM EDTA, 25 % (wt/vol) sucrose, pH 8.0). Then, cells were sonicated with a sonopuls HD 2070 homogenizer (Bandelin, Berlin, Germany) for 10 min (pulse: 30 s, pause: 30 s) at an amplitude of 40 %. The cell lysate was centrifuged for 1 h at 4 °C and 7000 g, the pellet was resuspended in washing buffer (10 mM Tris, 1 mM EDTA, pH 8.0), centrifuged again with the same settings and dissolved in solubilization buffer (20 mM Tris, 2 mM EDTA, 8 M urea, 100 mM glycine) at 4 °C under agitation overnight. Solubilized IBs were centrifuged for 30 min at 4 °C and 7000 g. The protein concentration of the supernatant was determined with a Nanodrop ND-1000 spectrophotometer (Peqlab Biotechnology GmbH, Erlangen, Germany) by measuring the absorbance (A) at 280 nm, and using the reference value at 10 g/l, $A^{1\%}_{\mathrm{OmpLA}} = 26.68$ (calculated from ProtParam, Expasy, the Swiss Bioinformatics Resource Portal). For blank measurement buffer was used.

Refolding of OmpLA was executed by adding the refolding buffer by drop dilution under agitation at 50 °C to reach final concentrations of 0.33 mg/mL OmpLA, 20 mM Tris, 2 mM EDTA, 0.80 M urea, 10 mM glycine and 35 mM LDAO (pH 8.3). The mixture was further agitated for 16 h at 50 °C. The refolded fraction was centrifuged for 15 min at 4 °C and 7000 g and filtered through a 450-nm poly (-vinylidene fluoride) filter (Carl Roth, Karlsruhe, Germany). The folded protein fraction was separated from the unfolded one by anion exchange chromatography on a 20 ml (4x5 ml) tandem HiTrap DEAE column (GE Healthcare, Solingen, Germany) in 20 mM Tris, 2 mM EDTA, 35 mM LDAO (pH 9.5) with a two-step gradient of 105 mM and 1.5 M KCl. The pooled folded fractions were dialyzed [20 mM Tris, 2 mM EDTA, 12 mM LDAO (pH 8.3)] overnight and concentrated on a 5 ml Resource Q column (GE Healthcare, Solingen, Germany) in 20 mM Tris, 2 mM EDTA and 12 mM LDAO (pH 8.3). The protein was eluted with 20 mM Tris, 2 mM EDTA, 12 mM LDAO and 1.5 mM KCl (pH 8.3) and further desalted on a PD-10 column (GE Healthcare, Solingen, Germany) using 20 mM Tris, 2 mM EDTA and 2 mM LDAO (pH 8.3). The final protein concentration was measured as described above.

Vesicle Preparation. Lipids (POPC, POPE, POPG) were dispersed in a 2:1 vol/vol chloroform/methanol mixture, dried under a stream of nitrogen and stored in vacuum overnight to ensure complete solvent evaporation. For lipid hydration the reconstitution buffer (20 mM Tris, 2 mM EDTA, pH 8.3) was added. The formation of lipid vesicles was achieved by intermittent vigorous vortexing at 15 °C > T_m (lipid melting temperature) for 1 h. Large Unilamellar Vesicles (LUVs) were prepared by 31-fold extrusion (mini extruder: Avanti Polar Lipids, Alabaster, AL, USA) through a polycarbonate filter (Whatman Nuclepore™ Track-Etched Membranes from Merck, Darmstadt, Germany) with a pore diameter of 100 nm. LUV formation was assisted by doping the bilayers with 5 mol% POPG (1). POPG does not affect membrane structure or protein activity at presently low concentrations (see below). Vesicle size was checked with dynamic light scattering (DLS) using a Zetasizer Nano ZSP (Malvern Panalytical, Malvern, UK). We found unimodal size distributions centered at $z_{av} = 133 \pm 12$ nm (Fig. S1).

Protein Reconstitution. For reconstitution, OmpLA solubilized in 2 mM LDAO was added drop by drop to the lipid vesicles at 35 °C and 350 rpm in a thermomixer (Eppendorf, Hamburg, Germany) to reach a final lipid/protein mole ratio of 300:1 (600:1 and 900:1, respectively). LDAO was removed by dialysis against 50 mM Tris, 2 mM EDTA and 200 mM NaCl (pH 8.3) overnight. The proteoliposomes were extruded 31 times through a 100 nm polycarbonate filter. The size of the proteoliposomes was determined by DLS to $z_{av}^{\mathrm{sym}} = 115 \pm 7$ nm, i.e. about equal to that of POPC LUVs (Fig. S1).

Preparation of Asymmetric Proteoliposomes. Asymmetric proteoliposomes were prepared following the heavy donor cyclodextrin exchange protocol (2); see Fig. S2 for a schematic. Acceptor and donor lipids were weighed (1:2 mol/mol). Acceptor vesicles were proteoliposomes (LUVs) with various lipid compositions (see Tab. S1). Donor lipids were POPE multilamellar vesicles (MLVs) in 20 wt% sucrose, obtained by 5 freeze/thaw cycles. Donor vesicles were diluted 20-fold with water and centrifuged at 20000 g for 30 min. The pellet was suspended in 35 mM mβCD (lipid:mβCD 1:8 mol/mol) and incubated for 2 h at 40 °C under agitation. Proteoliposomes were added and incubated for 30 min. The exchange was stopped by 8-fold dilution with water. The mixture was centrifuged at 20000 g for 30 min. The removal of residual cyclodextrin and sucrose was done by washing the sample three times with H_2O using 15 ml Amicon centrifuge filters (100 kDa cut-off, Merck, Darmstadt, Germany) at 5000 g. Asymmetric vesicles were concentrated to < 500 µl. To ensure the absence of donor MLVs the vesicle size

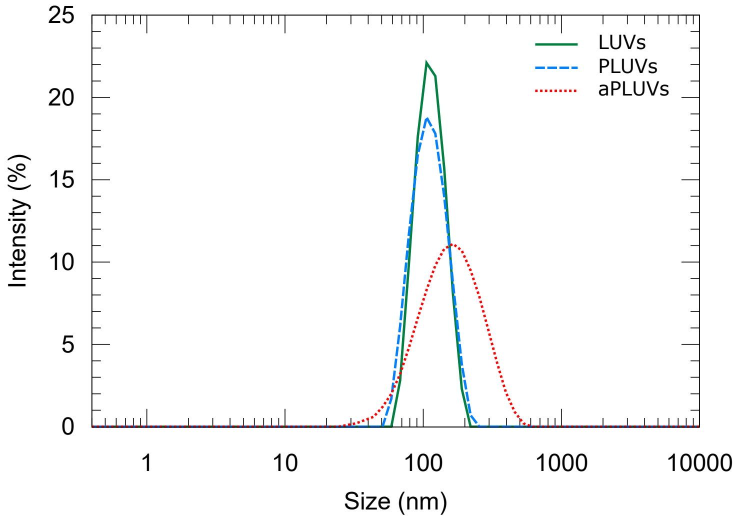

Fig. S1. Size distribution by intensity of symmetric LUVs (green line), symmetric (blue dashed line) and asymmetric (red dotted line) proteoliposomes.

was checked by DLS (Fig. S1). We observed slightly increased vesicle sizes and size distributions ($z_{av}^{\text{asym}} = 160 \pm 20$ nm), but no signs of contamination with smaller or larger aggregates.

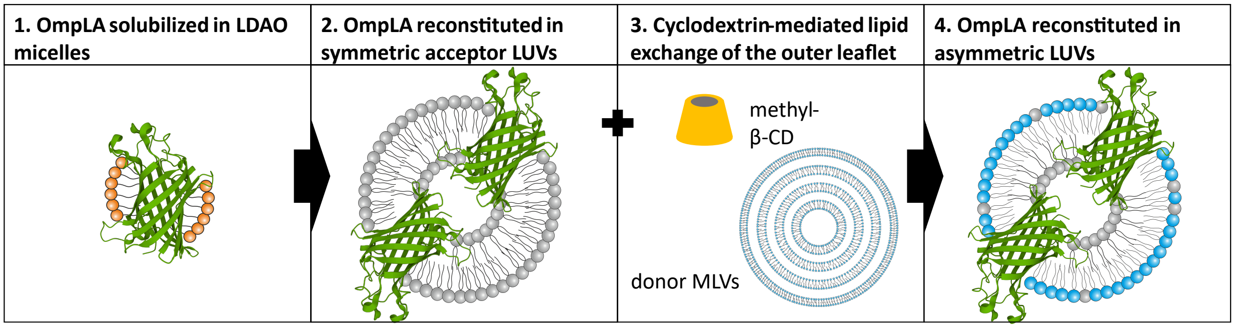

Fig. S2. Schematic of OmpLA reconstitution into LUVs and cyclodextrin-mediated outer leaflet lipid exchange (PDB code: 1QD5 (3)).

Phosphate Assay. The lipid samples were carbonized first in a heated metal block at maximum heat. After cooling, 0.4 ml acid mixture (9:1 vol/vol conc. H_2SO_4:$HClO_4$) was added and the sample was heated up again for 30 min. 9.6 ml of a reaction mixture (22 ml reagent A: 10.5 mM ANSA, 0.7 mM $Na_2S_2O_5$, 40 mM Na_2SO_3; 500 ml reagent B: ammonium heptamolybdate tetrahydrate (0.26 %)) was added to the cool samples. After mixing, the tubes were placed in a sand bath at 90 °C for 20 min. The extinction of the cooled samples was measured on a Spectrophotometer Onda V-10 Plus (Labbox Labware, S.L., Barcelona, Spain) at 830 nm. The phospholipid concentration was calculated by using a calibration curve (3.2 mM KH_2PO_4 as phosphate-standard-solution containing $1 - 14$ μg phosphor).

Colorimetric Enzyme Activity Assay. The thiol group of HEPC released upon hydrolysis reacts with DTNB by forming 2-nitro-5-thiobenzoate. Proteoliposomes with an OmpLA concentration of 40 µg/ml were incubated with 1 mM HEPC and 0.8 mM DTNB for 1 h in the dark at room temperature before $CaCl_2$ addition (final concentrations: 4 µg/ml OmpLA, 0.98 mM HEPC, 0.78 mM DTNB and 20 mM $CaCl_2$). Absorbance was monitored at 412 nm with a V-630 UV-Vis spectrophotometer (Jasco, Groß-Umstadt, Germany) using the time course measurement software. The measurement range was from 0 to 7320 seconds (after 120 seconds of measurement (baseline) $CaCl_2$ was added) with a data pitch of 5 seconds and a band with of 1.5 nm. The response was set "fast". A 105.251-QS glass cuvette (Hellma Analytics, Müllheim, Germany) with a volume of 60 μl and a thickness of 3 mm was used. The blank measurement was the empty light pathway. The normalized specific activity, $\bar{a}$, was calculated following

$$\overline{a} = \frac{\dot{A}_{412}}{\epsilon \; d \; c_{\mathrm{OmpLA}}}, \quad [1]$$

where $\dot{A}_{412}$ is the time-derivative of the absorbance at 412 nm (calculated in the linear range 5−20, 5−30, 5−80 min for [OmpLA]/[lipid] = 1:300, 1:600, 1:900, respectively), ϵ=13.6 $mM^{-1}cm^{-1}$ is the molar extinction coefficient of 2-nitro-5-thiobenzoate at 412 nm (4), d is the optical pathlength, and c_{OmpLA} is the concentration of refolded OmpLA. Figure S3 shows the results of this assay for POPC proteoliposomes. The specific activity of OmpLA increased linearly with protein concentration.

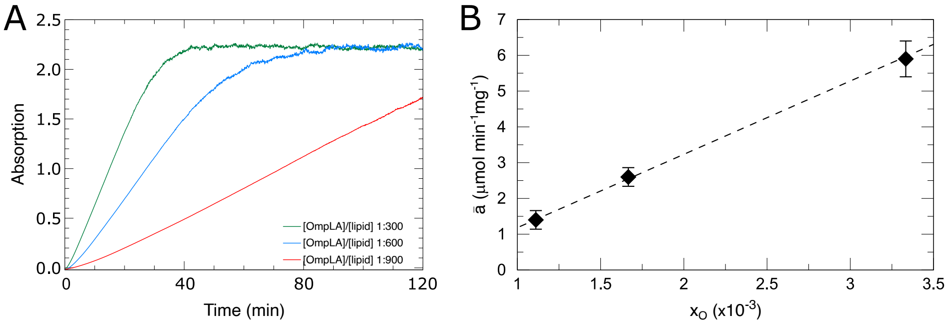

Fig. S3. Colorimetric OmpLA activity measurements in POPC proteoliposomes. Panel A shows time traces at different [OmpLA]/[lipid] molar ratios: 1:300 (green), 1:600 (blue), and 1:900 (red). Panel B displays the determined specific activities, revealing a linear increase with protein molar ratios x_O = [OmpLA]/[lipid].

High Performance Thin Layer Chromatography (HPTLC). The enzymatic degradation of the proteoliposomes was determined by TLC. After lipid extraction against organic solvent (2:1 vol/vol chloroform/methanol) based on the Folch extraction method (5), the samples were spotted on a silica plate (Sigma-Aldrich, Steinheim, Germany) with the automatic TLC sampler 4 (CAMAG, Muttenz, Switzerland). The mobile phase in the developing chamber was a solvent mixture composed of 32.5:12.5:2 vol/vol/vol $CHCl_3$/MeOH/H_2O. After drying, the plate was immersed in a developing bath (5.08 g $MnCl_2$ dissolved in 480 ml H_2O, 480 ml EtOH and 32 ml H_2SO_4), which is sensitive to double bonds, and dried for 15 min at 120 °C (6). To quantify the lipid concentrations the plate was scanned with the TLC scanner 3 (CAMAG, Muttenz, Switzerland) and further analyzed with WinCats software.

Results of time resoled HPTLC experiments are shown in the main document. Fig. S4 shows the results for POPG, which was used to aid the formation of LUVs for protein reconstitution (see above). No significant changes of POPG levels were observed during the experiments.

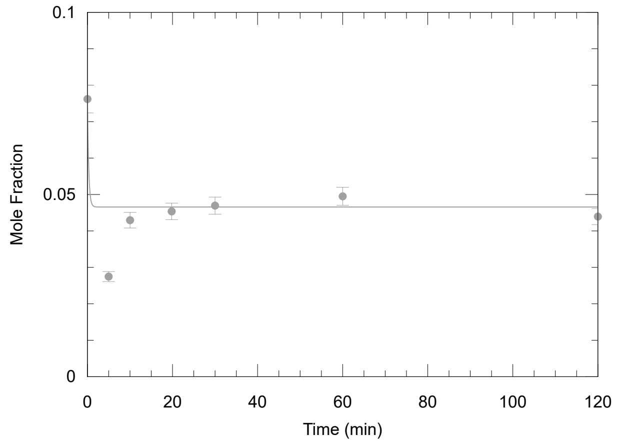

Fig. S4. Evolution of POPG levels in POPC proteoliposomes.

Ultra Performance Liquid Chromatography Mass Spectrometry (UPLC-MS). Samples were dissolved in 300 μl 30:15:5 vol/vol/vol isopropanol/methanol/water . Chromatographic separation was performed using a 1290-UHPLC system (Agilent, Waldbronn, Germany) equipped with a BEH-C18-column, 2.1×150 mm, 1.7 μm (Waters, Manchester, UK). The autosampler compartment was set to 8 °C and 1 μl sample was injected. A binary gradient was applied. Solvent A was water, solvent B was 2-propanol. Both solvents contained phosphoric acid (8 μM), ammonium acetate (10 mM) and formic acid (0.1 vol%). The linear gradient started at 50 % solvent B at a constant flow rate of 0.15 ml/min and increased to 100 % solvent B within 20 min. In the

following 4 min solvent B percentage was kept at 100 %. The column was re-equilibrated for 5 min, resulting in a total HPLC run time of 30 min. The column compartment was kept at 50 °C. A 4670 triple quadrupole mass spectrometer (Agilent, Waldbronn, Germany) equipped with an ESI source was used for analysis. The following source parameters were used: source temperature 300 °C, sheath gas (N_2) temperature: 400 °C. The capillary voltage was 3.5 kV in positive ionization mode. Samples were analyzed in MRM mode and data analysis was done using the MassHunter 10.0 software package (Agilent, Waldbronn, Germany).

Figure S5 shows the analysis of POPC/POPE proteoliposomes. Note that the instrument was not calibrated for quantitative concentration measurements. Different levels of lipid concentration between different lipid species hence do not reflect actual amounts present.

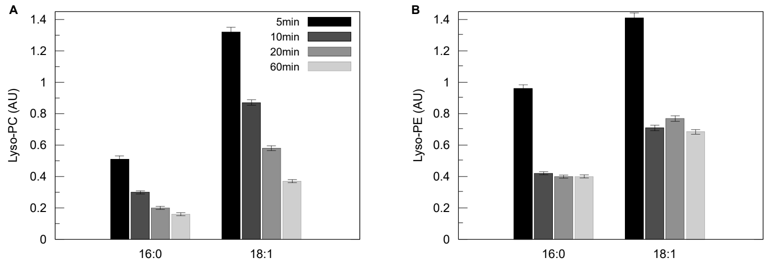

Fig. S5. Hydrolysis products of sym_1 detected by UPLC-MS: 16:0 lyso-PC and 18:1 lyso-PC in (A); 16:0 lyso-PE and 18:1 lyso-PE in (B).

Supporting Tables

Table S1. Lipid composition of studied asymmetric proteoliposomes.

System	acceptor LUV compositon (mol%)			aPLUV composition (mol%)			C_{lipid} (mM)	C_{OmpLA} (μM)
	POPC	POPE	POPG	POPC	POPE	POPG		
asym_1	95	0	5	53 (51)	42 (44)	5 (5)	1.03 (1.51)	4.6 (4.6)
asym_2	80	15	5	52 (52)	43 (43)	5 (5)	1.09 (0.94)	13.8 (14.1)
asym_3	70	25	5	43 (46)	52 (49)	5 (5)	0.65 (0.82)	8.2 (8.9)
asym_4	60	35	5	44 (42)	51 (53)	5 (5)	0.36 (0.51)	1.3 (4.6)

[a] Lipid and protein concentrations were measured after lipid exchange; [b] Numbers in brackets are derived from an equivalent repetitive measurement.

Table S2. Lipid composition of studied symmetric proteoliposomes.

System	LUV composition (mol%)			C_{lipid} (mM)	C_{OmpLA} (μM)
	POPC	POPE	POPG		
sym_{POPC}	95 (95)	-	5 (5)	1.92 (1.89)	15.6 (14.9)
sym_1	50 (49)	45 (46)	5 (5)	1.31 (1.25)	7.3 (7.9)
sym_4	41 (43)	54 (52)	5 (5)	1.24 (1.51)	7.9 (7.9)
sym_{POPE}	-	90 (90)	10 (10)	0.91 (0.90)	7.3 (7.6)

[a] Symmetric LUVs were prepared based on the composition of asymmetric proteoliposomes (Tab. S1).; [b] Lipid and protein concentrations were measured after lipid exchange. Time rates and turnover numbers are fitting results; [c] Numbers in brackets are derived from an equivalent repetitive measurement.

Table S3. Lipid hydrolysis-rates and turnovers in symmetric proteoliposomes.

System	$\bar{k}_i$ (s^{-1})			Δ $[lipid]/[lipid]$	
	POPC	18:1 lyso-PC	POPE	POPC	POPE
sym_{POPC}	0.71 (0.75)	0.16 (-)	-	0.20 (0.24)	-
sym_1	0.53 (0.49)	0.90 (0.88)	0.61 (0.66)	0.39 (0.38)	0.25 (0.31)
sym_4	0.65 (0.87)	0.48 (1.01)	0.33 (0.20)	0.41 (0.35)	0.21 (0.16)
sym_{POPE}	-	-	0.96 (1.62)	-	0.29 (0.39)

[a] Numbers in brackets are derived from an equivalent repetitive measurement; [b] Relative errors associated to the fitting parameters are about 10−20% for hydrolysis rates and 2% for turnover values.

Table S4. Lipid hydrolysis-rates and turnovers in asymmetric proteoliposomes.

System	$\bar{k}_i$ (s^{-1})			Δ $[lipid]/[lipid]$	
	POPC	18:1 lyso-PC	POPE	POPC	POPE
asym_1	0.08 (0.08)	0.68 (0.45)	0.10 (0.06)	0.97 (0.96)	0.87 (0.89)
asym_2	0.04 (0.05)	0.44 (0.80)	0.06 (0.06)	0.98 (0.97)	0.86 (0.90)
asym_3	0.004 (0.004)	0.13 (0.11)	0.014 (0.016)	0.75 (0.97)	0.87 (1.00)
asym_4	0.21 (0.06)	3.9 (0.45)	0.23 (0.07)	0.93 (0.93)	0.97 (0.95)

[a] Numbers in brackets are derived from an equivalent repetitive measurement; [b] Relative errors associated to the fitting parameters are about 20% for hydrolysis rates and 3% for turnover values.

Table S5. Hydrolysis rates of POPC in sn-1 position, k_1, after OmpLA activation with $CaCl_2$.

Sample	$\bar{k}_1$ (s^{-1})
$asym_1$	0.010 (0.009)
$asym_2$	0.005 (0.006)
$asym_3$	0.001 (0.001)
$asym_4$	0.03 (0.006)
sym_1	0.06 (0.06)
sym_4	0.08 (0.106)

Numbers in brackets are derived from an equivalent repetitive measurement. The parameter k_1 presents only qualitative values due to overparameterization.

Supporting Figures

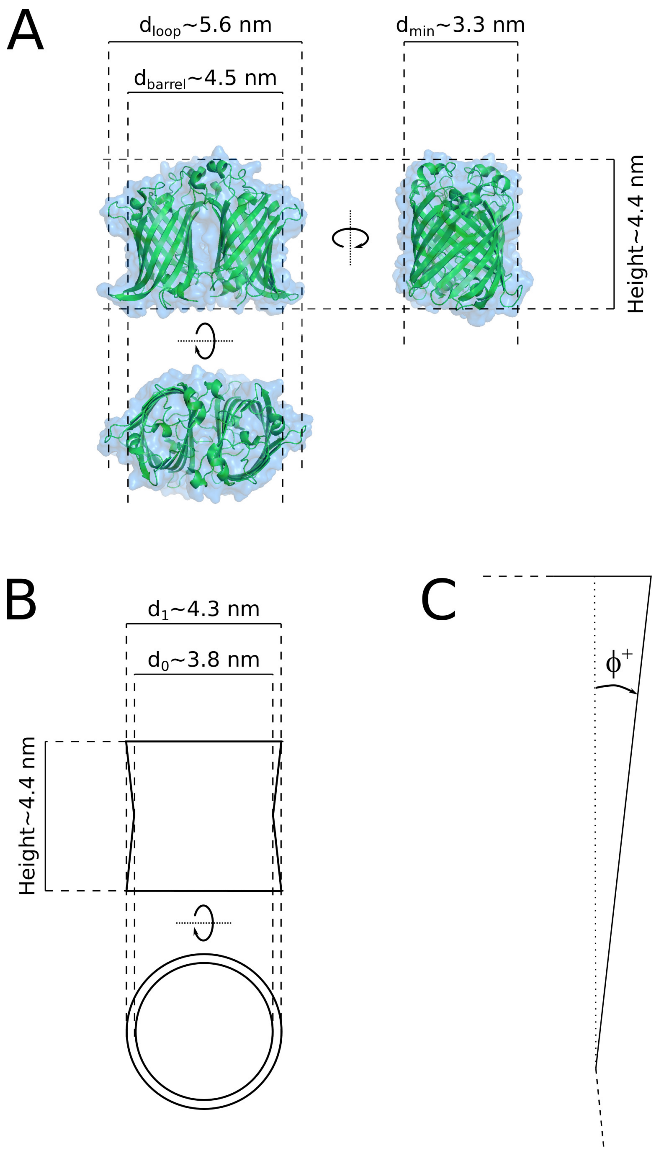

Fig. S6. A) Average length values of an OmpLA dimer. The cartoon (green) and surface (transparent blue) were obtained using PyMol (PDB code: 1QD6 (3)). B) Hourglass approximation of an OmpLA dimer. The minimum, $d_0 = 2R_0$ and maximum diameter, d_1, of the ideal hourglass with circular cross-section were calculated by approximating the actual cross-section of the dimer as an ellipsoid and conserving the area: $d_0 = \sqrt{d_{\text{barrel}} d_{\text{min}}}$ and $d_1 = \sqrt{d_{\text{loop}} d_{\text{min}}}$. C) The angle ϕ was calculated as $\tan\phi = \frac{d_1/2 - R_0}{Height/2}$.

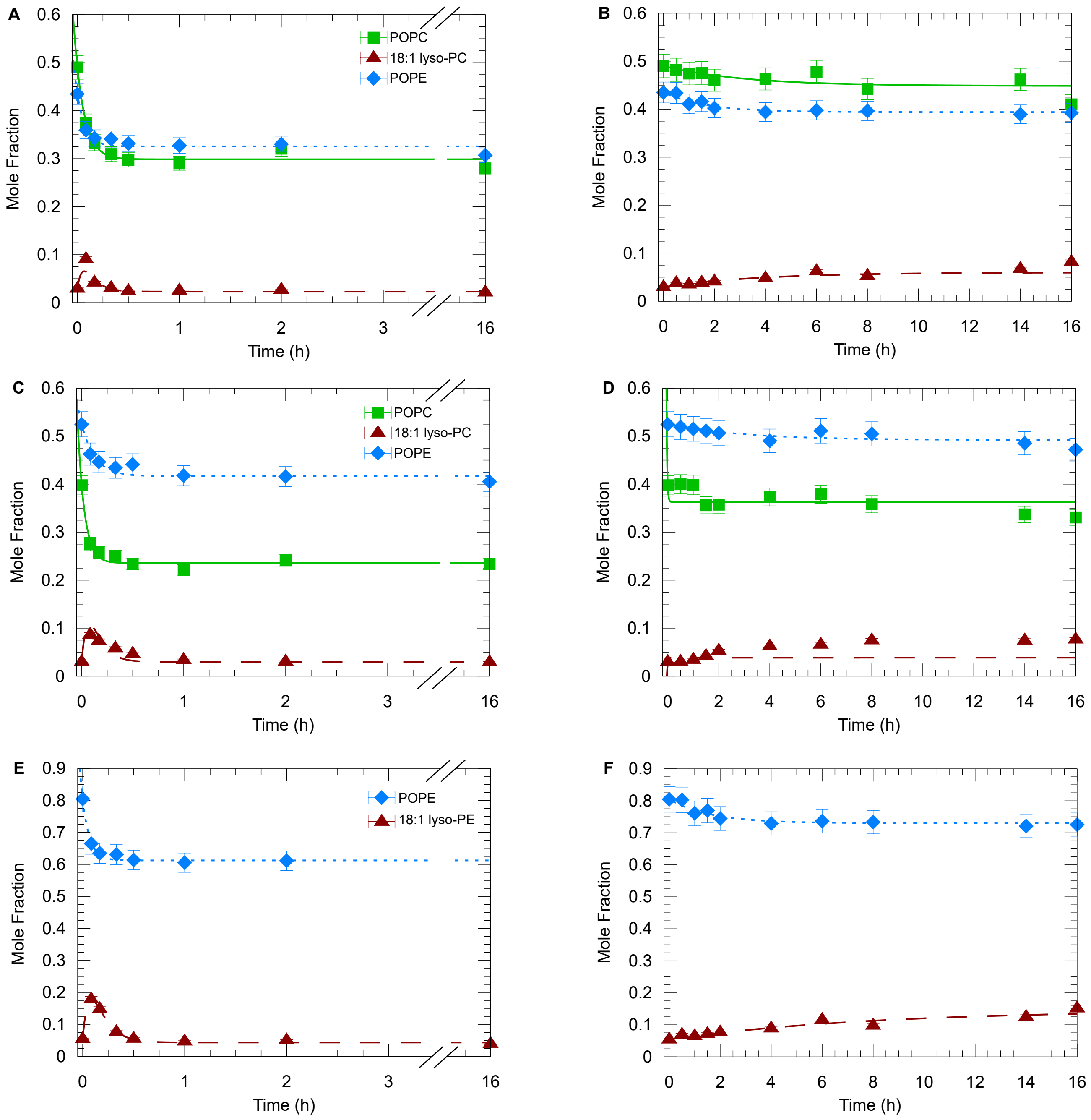

Fig. S7. Hydrolysis of symmetric proteoliposomes sym_1 (A), sym_4 (C) and sym_{POPE} (E) upon OmpLA activation with $CaCl_2$ and its basal activity (B,D,F).

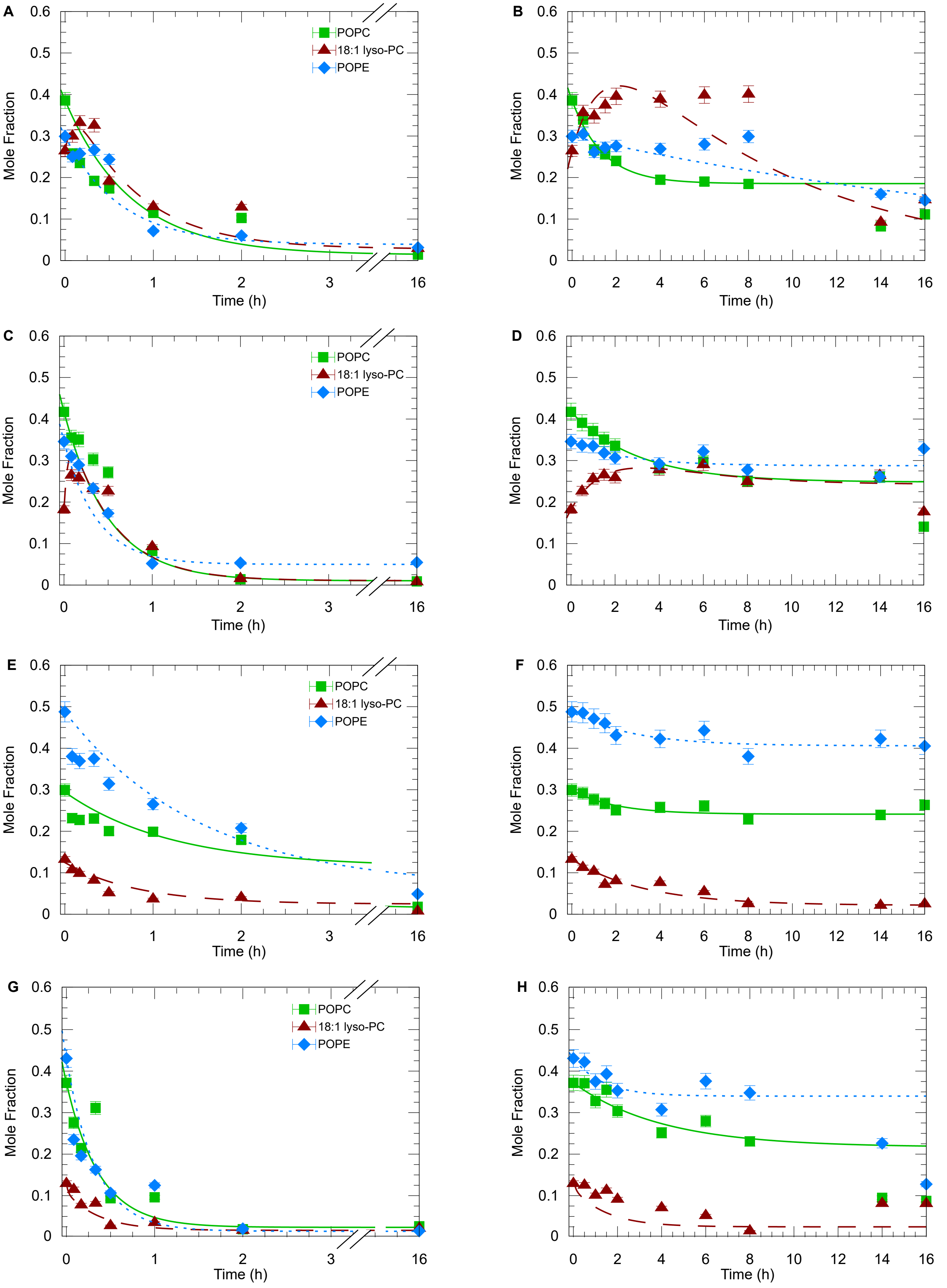

Fig. S8. Hydrolysis of asymmetric proteoliposomes asym$_1$ (A), asym$_2$ (C), asym$_3$ (E), asym$_4$ (G) upon OmpLA activation with $CaCl_2$ and its basal activity (B,D,F,H).

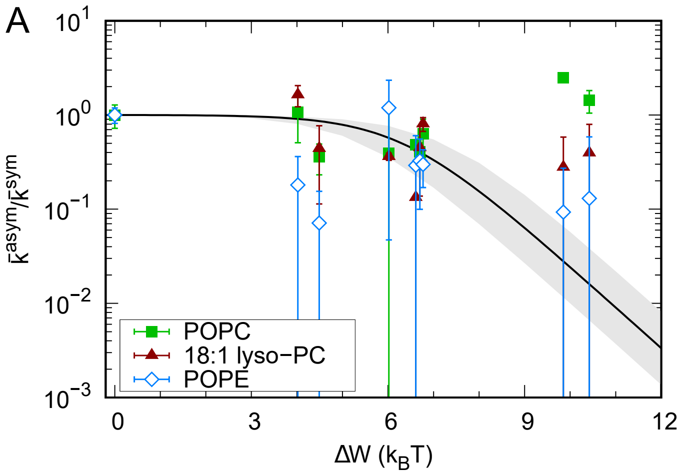

Fig. S9. Ratio of normalized hydrolysis rates, $\bar{k}_i$, between asymmetric and symmetric proteoliposomes: POPC (squares), 18:1 lyso-PC (triangles), and POPE (diamonds). The curve shows a global fit based on Eq. 4, and the gray band depicts the 99%-confidence interval. Fitting result: $\Delta G^{*,\mathrm{sym}} = -6.3 \pm 0.3\ k_B T$. Reference rates at $\Delta W \simeq 0$ are the averages from symmetric proteoliposomes at different POPC/POPE ratios (see Table 1). Uncertainties for ΔW are in the range 20−40%.

References

1. B Eicher, et al., Intrinsic curvature-mediated transbilayer coupling in asymmetric lipid vesicles. *Biophys J* **114**, 146–157 (2018).
2. M Doktorova, et al., Preparation of asymmetric phospholipid vesicles for use as cell membrane models. *Nat Protoc* **13**, 2086–2101 (2018).
3. HJ Snijder, et al., Structural evidence for dimerization-regulated activation of an integral membrane phospholipase. *Nature* **401**, 717–721 (1999).
4. GL Ellmann, Tissue sulfhydryl groups. *Arch Biochem. Biophys* **82**, 70–77 (1959).
5. J Folch, M Lees, GH Sloane Stanley, , et al., A simple method for the isolation and purification of total lipids from animal tissues. *J biol Chem* **226**, 497–509 (1957).
6. OL Knittelfelder, SD Kohlwein, Thin-layer chromatography to separate phospholipids and neutral lipids from yeast. *Cold Spring Harb Protoc* **2017** (2017).
